# Class-Room Talks

**Published:** 1887-12

**Authors:** J. T. Kent


### CLASS-ROOM TALKS.

PROFESSOR J. T. KENT, M. D.

Chronic tendency to congestion of the head, when Bell. has been the remedy that gave relief to the acute expression of the disease, Calc. Now, I don't mean you to understand that during the attack Calc. would be the better remedy. Bell. corresponds more fully to the acute manifestation. Calc. would *agg.* too strongly; but after the attack a dose of Calc. will cure the tendency to repeated return of these congestive conditions.

So when each time the patient takes a cold, he has swollen tonsils, tonsilitis, and has chronic induration of the tonsils—Baryt. c.

Now, we do not mean that Bar. c. would be the best indicated remedy during the acute attack—many remedies may be better indicated—but that a dose of Bar. c., *after* the attack, *would* be indicated, and would cure the tendency to return.

*Don't commence the treatment of any chronic disease during the exacerbations.*

In epilepsy you will never cure unless you first find a remedy that covers and corresponds in every respect to the acute attack. Then follow with the complementary or chronic as the curative.

In chills and fever a prescription before or during the paroxysm will *certainly* increase the violence of the paroxysm, and hinder, if not complicate, matters.

---

Hahnemann has been accused of alternation, of saying that Bry. and Rhus alternated.

Now, Hahnemann did not mean you were to put one remedy in one glass and one in another, giving first of one and then the other: Bry. and Rhus are complements of one another, and Hahnemann meant just this: You have had the symptoms and given the similar, Bry., and you will often find that when Bry.

has ceased its action the symptoms of Rhus will begin to shadow forth. Now wait a little; you will have a clear picture of Rhus. You give it, and after a little Rhus will have done its work. Again the symptoms of Bry. may appear, and so on until you have finished your case.

Arn., Rhus, and Calc. often follow one another *this* way: A sprain in joint, bruised condition of muscles, would be well covered immediately by Arn. The injury does well for a time, but after a week or two there is still some weakness and pain. Now Rhus is also similar, but belongs to a later period. So Rhus takes up the case, carries him comfortably on for some months, when he suddenly finds its power over the condition gone, and that he has a rheumatic stiffness in the strained joint coming on after *cold, damp* weather.

Now Calc. is indicated and will finish the case.

In such conditions the needed remedy seems to go deeper and deeper, and to eradicate the remaining vestiges of excited psora.

Hahnemann has said that we would often find that certain of the remedies rotated, *i. e.*, Sulph., Calc., Lyc., one might say of that, as of alternation, to place each in a tumbler by the bedside, giving from first, second, and third in succession, etc.; but that is not the point. The great master intended you to know that many times (not always) the symptoms of Sulph. would be followed by those of Calc., and those again by symptoms of Lyc., returning to Sulph. after Lyc., and so on until the case is completed. It is well for you to know these things, that you may be watchful and prepared to solve the problems as they arise.

The better prescribers use the most profound reasoning in the study of their cases and in their search for a remedy. To show you how you must think and study out your symptoms—by a comparatively simple case—and how to prescribe when you seemingly have but one symptom:

A lady comes to my office with extreme restlessness of lower extremities. Well, I think, that is Zinc., pre-eminently, and many others. Yet I do not stop there. I inquire further, and find that a few days before she has been out in the rain and got wet. "Where, your feet?" "Oh! no! My feet were protected, but my head got very wet." Why, think I, that sounds like Bell. I must see if Bell. has restlessness of the limbs. Sure enough, Bell. has it, and Bell. cures with no further return of symptoms.

